# Optimizing the first-line treatment for metastatic colorectal cancer

**DOI:** 10.3389/fonc.2023.1246716

**Published:** 2023-10-16

**Authors:** Sara Cherri, Ester Oneda, Laura Zanotti, Alberto Zaniboni

**Affiliations:** Department of Clinical Oncology, Fondazione Poliambulanza, Brescia, Italy

**Keywords:** coloncancer, chemotherapy, metastatic cancer, first line, target therapy

## Abstract

Colorectal cancer represents an important oncological challenge both for its incidence, which makes it an important health problem, and for its biological complexity, which has made clinical results very difficult in terms of outcome for this category of patients. To date these diseases should not be treated as a single entity but it is necessary to distinguish colorectal cancers based on characteristics that nowadays are essential to have greater therapeutic benefits. These include the sideness of the disease, the state of microsatellites, the presence of prognostic and predictive mutations of response to treatments currently available in clinical practice, which are associated with new therapeutic targets. The greatest challenge in the future will be to circumvent the resistance mechanisms that make this disease very difficult to treat with good long-term results by studying effective combination treatments with a good toxicity profile. Once such combinations or targeted treatments are consolidated, it will be desirable to shift the best therapies to the first line treatment to make them immediately accessible to the patient. It will also be essential to refine the selection of patients who can benefit from these treatments.

## 1 Introduction

In recent years, there has been a remarkable rapid change in clinical practice in Medical Oncology thanks to the introduction of innovative drugs that have rapidly moved to the first line treatment of cancer patients suffering from metastatic disease, especially as regards tumors such as lung cancer, breast cancer, kidney cancer and melanoma. This happened thanks to the greater knowledge gained in the field of cancer molecular biology which led to the introduction of target drugs. As far as patients with metastatic colorectal cancer (mCRC) are concerned, this “revolution” has had a much less felt impact, partly due to the complexity of the molecular biology of this pathology, and partly due to the small number of patients to date identifiable for targeted treatments and with less encouraging results than other types of cancer. To better catalog the types of colorectal cancer (CRC) and consequently the therapeutic possibilities, different models have been proposed to identify subtypes of CRC based on large-scale gene expression studies. The most validated model to date is the one proposed by the International Consortium, i.e. the consensus molecular subtypes (CMS), which identify four distinct types of CRC on the basis of mass transcriptomic signatures: CMS1 (immune MSI), CMS2 (canonical), CMS3 (metabolic) and CMS4 (mesenchymal) (1). However, to date, this classification cannot be used in clinical practice because it does not translate into a different therapeutic approach, probably because CMS fails to reflect the biological complexity of colorectal cancer. It is essential to identify new biomarkers to improve the therapeutic approach and sequential strategies of colorectal cancer. The current indications of the first line of metastatic colorectal cancer (mCRC) will be discussed below along with the data of the main studies in progress and the potential future therapeutics approaches. The limitations of current 1st line treatments represent the main starting point for the development of new treatment options. The molecular heterogeneity that characterizes colorectal tumors is the main cause of the mechanisms of resistance to treatments and this assumption has favored the study of combined treatments with the aim of eluding both acquired and intrinsic resistance, blocking multiple signal pathways implicated in the mechanisms of carcinogenesis. However, the limitation of treatments involving the combination of target molecules is the cumulative toxicity. A possible solution could be to observe the possible development of resistance during treatment by monitoring the mutational status of the disease with the aid of next generation sequencing (NGS). The identification of mutations in progress could potentially anticipate disease progression and suggest the combination of target treatments in selected patients. There are several studies, involving different molecules and different oncological pathologies, which are trying to validate an algorithm applicable in clinical practice (2). Considering that MSS and MSI-H mCRC tumors are two different entities of the same disease that differ both in etiologic, clinical, pathological and treatment outcome characteristics, the first step is to divide the stable disease from the unstable one. To this reading must be added, especially in patients with microsatellite stability, the knowledge of the mutational status of KRAS and BRAF. Further pieces are gradually being added with the greater biological knowledge of these pathologies, such as the presence of other mutations.

## 2 First line therapy

**Figure 1:**
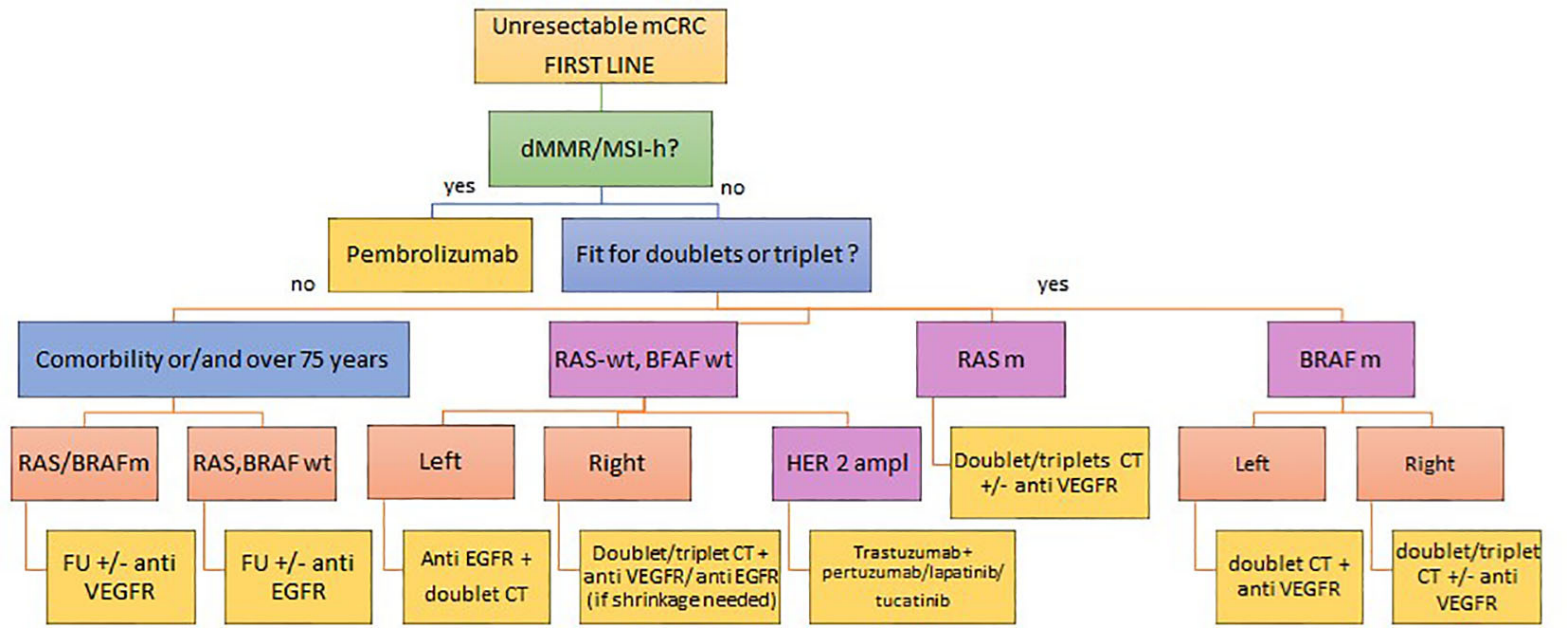
Current therapeutic algorithm for first line mCRC.

At present, the first step in choosing the first-line treatment must fundamentally take into account the following relevant elements, such as the characteristics of the disease itself, both in terms of molecular biology (mutations for KRAS, NRAS, BRAF, microsatellite status), the disease burden, the sideness (right and left colon) and the clinical presentation at onset, as well as the patient-related factors, especially performance status, age and comorbidities. At the present time, a consolidated practice for more than a decade and confirmed by various molecular studies, it is the sine qua non condition for starting treatments through the molecular profiling of KRAS, NRAS and BRAF. The baggage of knowledge in the following years has led to a better knowledge on the complexity and heterogeneity of the disease, adding further building blocks for therapeutic decision making in the first-line treatment of mCRC such as the importance of the side of the primary tumor and the state of instability of microsatellites (See **Figure 1**).

### 2.1 How to choose first line therapy for MSS mCRC patients

The backbone of first line therapy for MSS mCRC is the association of fluoropyrimidine, oxaliplatin and/or irinotecan. These drugs can be use as monotherapy (capecitabine), in doublet or triplet associated with biological drugs (anti EGFR or anti VEGFR). The first data to consider in choosing the treatment is the clinical presentation of the patient affected by mCRC, which is a mandatory information to decide for intensive treatments. The second step is the consideration of the sideness of the primitive which is fundamental in the correct reading of the molecular profile of the disease, which represents the third important information that guides the choice.

#### 2.1.1 Clinical presentation of the mCRC patient candidate for first line treatment

Clinical evaluation cannot ignore the choice of the first-line treatment, remaining today the most reliable guide in therapeutic decision-making. This assessment must take account of age, any comorbidities and performance status.

Of these factors, performance status remains the most important parameter. Several studies have evaluated the expected response to treatment in relation to performance status at disease onset. The performance status (PS) is commonly evaluated according to the numerical scale of the Eastern Cooperative Oncology Group (ECOG) which is divided into four levels based on the patient’s ability to take care of himself, to live daily activity including physical activities such as walking and working. Most clinical studies do not include patients with ECOG PS > or = 2.

Although a certain benefit of the treatments can also be demonstrated in categories of patients with poor PS (ECOG PS 2) and very poor PS (ECOG PS >2) (3) any proposal for chemotherapy treatments should be considered very cautiously given the potential toxicity. A study published in 2009 by Sargent and colleagues included over 6,000 mCRC patients who were candidates for first-line chemotherapy in 9 clinical trials. Patients with ECOG PS 2 had a higher rate of chemotherapy toxicity and higher 60-day all-cause mortality than patients with ECOG PS 0-1. Furthermore, ECOG PS2 was found to be prognostic for PFS, OS, and response rate (RR) (4). Differences in treatment toxicity and outcomes in terms of OS and PFS were found in a study analyzing patients with ECOG PS < 2 on a pooled data set from five clinical trials of 5FU-based treatment for metastatic colorectal cancer. These differences are even detectable between patients with ECOG PS 0 and patients with ECOG PS 1, with a more detrimental data of the latter, although many studies tend to compare these two categories of patients (5).

NCCN guidelines recommend identifying patients who are candidates for intensive cancer treatments compared to patients who are not due to clinical frailty, whether determined by age, comorbidity, or performance status. For the former, it will be feasible with doublet fluorouracil-based chemotherapy with a biological drug (anti VEGF or anti EGFR depending on the molecular profile and sideness), for the latter instead mono-chemo therapies based on fluorouracil with a biological drug should be favored (6). Clinical trials are conducted in an increasingly personalized way to consolidate clinical indications that favor correct treatment together with the preservation of a good quality of life in frail patients (7–9).

#### 2.1.2 Sideness of the tumour

A consolidated concept on the biological knowledge of colorectal tumors is the diversity between left side and right-side colon tumors which translates into a different behavior of these two pathologies, in terms of different prognosis and therapeutic proposal (10). Considering the different prognosis of patients affected by mCRC based on the sideness of the disease is essential to propose the correct first line treatment (11). This diversity derives from complex factors, not yet fully known, but it is certainly partly explained by different risk factors, by a different mutational profile and by a different genetic origin, in particular, posterior intestine for the left colon (intended from the splenic flexure to the rectum) and embryonic midgut for the right colon (intended from the hepatic flexure to the cecum). The first interesting data on the differences between these two pathologies date back to about 20 years ago. A study published by Glebov and colleagues in 2003 evaluated the difference between the gene signatures of right and left colorectal cancer and reported more than 1,000 differentially expressed genes between right and left colon, shedding light on the genetic complexity that characterizes colorectal cancer (12) and giving rise to numerous subsequent studies that have deepened this topic (1, 13, 14).

These differences translate into a different response to 1st line target treatments, already from the first studies of chemotherapy in association with Cetuximab, an anti EGFR monoclonal antibody, it was evident that the localization of primary tumors on the left RAS Wild type must be considered a predictor of response (15, 16). Therefore, in patients affected by mCRC of the left colon the recommendations of the international guidelines are clear, i.e., in RAS WT diseases the cornerstone therapy is based on the fluorouracil-based doublet with anti EGFR antibody, in the disease with RAS mutation chemotherapy is recommended (double or triple) + the association with the anti-VEGF. On the other hand, RAS WT right side colorectal cancers have a lower probability of responding to the anti EGFR antibody; therefore, the cornerstone of the first line of treatment remains the double or triple of chemotherapy + anti-VEGF antibody. It should be noted that data on the transverse colon are very scarce and to date the correct definition of the best first line treatment for this category of patients is still undefined.

#### 2.1.3 Molecular profile

As previously mentioned, the reading of the molecular data in the first-line of metastatic colorectal cancer disease must consider the sideness of the tumor, the patient’s clinic, and his performance status. The different molecular profiles, their prognostic and predictive significance, and current first-line indications for MSS mCRC patients are reported below.

##### 2.1.3.1 RAS WT BRAF WT

This category of patients is a candidate for chemotherapy associated with anti EGFR monoclonal antibody.

The first data on the importance of selecting patients with RAS WT status date back to 2006 and derive from a small series of 30 patients with mCRC treated with Cetuximab, an anti EGFR monoclonal antibody, and screened for the KRAS mutation (17). Despite the small number of cases, the information deriving from this study was extremely significant, namely, patients with the KRAS mutation presented resistance to treatment with Cetuximab, suggesting the need for a greater selection of patients who were candidates for treatment, starting a major precision medicine research.

The OPUS and CRYSTAL trials evaluated the benefit of adding cetuximab in patients with metastatic colorectal cancer (mCRC) to the FOLFOX4 and FOLFIRI regimens, respectively, both concluding with the same postulate, specifically that patients with KRAS exon 2 tumor mutations do not show any benefit from adding cetuximab to the chemotherapy regimen unlike patients with wild type KRAS (18, 19). Similar were the conclusions of the phase III study for Panitumumab, a second anti EGFR drug approved in clinical practice, in combination with FOLFOX (20).

At present, knowledge on the biological complexity of colorectal tumors suggests that RAS status alone is not sufficient to select patients more likely to respond to anti-EGFR therapy and that probably despite RAS WT status, the presence of other mutations or losses of genes (such as BRAF, PTEN, EGFR) can affect the lack of response to treatment. The most probable scenario for the near future will be that of a genetic mapping capable of guiding an increasingly correct therapeutic choice (21).

##### 2.1.3.2 RAS mutated BRAF WT

In this category of patients, the cornerstone of treatment is represented by the combination of chemotherapy (doublet or triplet fluorouracil-based chemotherapy) with Bevacizumab, an anti-VEGF drug. To date we know that probably not all KRAS mutations have the same significance and that some mutations could have a greater prognostic relevance, in particular the presence of the KRAS G12C mutation has been associated with a worse survival than other KRAS mutations (22). It is known that colorectal tumors are the result of the progressive accumulation of numerous alterations of genes involved in the mechanisms of cell proliferation, differentiation, and cell survival. Being the KRAS oncoprotein crucially involved in the signaling cascade activated by the anti-epidermal growth factor receptor (EGFR), its pathological activation leads to a constitutive alteration of the signal transduction mechanisms downstream of EGFR with consequent resistance to treatments with anti-EGFR monoclonal antibodies such as Cetuximab and Panitumumab. For years, therefore, the presence of the KRAS mutation was considered a predictive factor of unresponsive for anti EGFR antibodies but considered non-targetable as previous attempts to target drugs on KRAS had given disappointing results. Over time, greater knowledge of this mutation has enriched its significance both in prognostic terms and in terms of response predictions to target drugs on the KRAS mutation.

###### 2.1.3.2.1 Future scenario

If until recently the KRAS mutation was considered un-targetable, the recent introduction of specific inhibitors of the KRAS G12C mutation in patients with advanced lung cancer has been followed with great enthusiasm, considering that about half of patients with mCRC has a KRAS mutation. However, this enthusiasm has subsequently decreased, considering that the KRAS G12C mutation is not the most frequent one for mCRC patients and considering that the response to treatment and overall survival data for KRAS G12C inhibitors in latter line turned out to be less exciting in tumors with mCRC than in lung cancer. It was seen in the CodeBreaK100 study (NCT03600883), heavily pretreated patients with colorectal cancer with the KRASG12C mutation were treated with at least one dose of sotorasib, the objective response was of 9.7%, very less impactful than observed in lung tumors (23). Among the combination studies the most promising are those in combination with anti EGFR monoclonal antibodies as the mutated KRAS G12C mCRC disease retains sensitivity to upstream EGFR signaling, thus leading to an adaptive resistance mechanism and consequent limited efficacy of inhibition of EGFR. KRAS for EGFR reactivation. In the phase Ib CodeBreaK 101 study of 40 mCRC pretreated patients with mutant KRASG12C received the combination of Sotorasib and Panitumumab, there was an ORR of 30% with a DCR of 93% (24). Similar published results from the phase I/II KRYSTAL-1 study in patients treated with the combination Adagrasib plus Cetuximab, which concluded with an ORR of 46% and a DCR was 100% (25). At ASCO 2023, the results of the phase Ib CODEBREAK101 (NCT04185883) study were presented, which evaluated the combination sotorasib + panitumumab + folfiri with an ORR of 58.1% and a good safety profile (26), which will be a further step towards understanding the role of addition of chemotherapy in these patients.

Numerous other combinations are being studied in combination with KRAS inhibitors such as MEK inhibitors, multikinase inhibitors, SOS1 inhibitors, PI3K inhibitors and mTOR inhibitors, with the aim of preventing adaptive resistance (27–30). It will be essential to collect data on the toxicity profile of such combinations and once again to understand how to select patients who may benefit from them. The data from these studies are still very immature, however phase III KRYSTAL-10 studies are underway for the combinations of KRAS inhibitors and anti EGFR monocolonal antibodies, currently from the second line of treatment (NCT04793958). However, given the very encouraging preliminary data, they could in the not very near future become 1st line standard in patients with mutant KRASG12C mCRC.

##### 2.1.3.3 RAS WT BRAF mutated

BRAF mutation represent the 10% of mutated CRC and has not only a prognostic value but also a predictive data of response to specific target therapy (31). Regarding the prognostic data, patients with BRAF V600E mutation have a worse prognosis and median overall survival than non-mutated patients (32). The development of a targeted treatment for this category of patients has helped add an important piece of treatment for patients with mCRC. In the phase III BEACON study, encorafenib, a BRAF V600E inhibitor, was combined with binimetinib, a MEK1/2 inhibitor, and cetuximab, showing an improvement in overall survival in both the triplet arm (cetuximab, binimetinib and encorafenib) and in the doublet arm (cetuximab and ecorafenib) (33). However, these data are to be reported after a first line of treatment in the metastatic setting, therefore currently the first line indications of patients with BRAF V600E mutated mCRC are mostly based on retrospective data collected from the main first line clinical trials for patients with mCRC. In fact, there are no randomized studies that have evaluated the added value of monoclonal antibodies, both anti VEGF and anti EGFR, in combination with standard 1st line chemotherapy (doublet or triplet). A very small subgroup of patients with BRAF V600E mutation (28 patients) is included in the phase III TRIBE study, therefore no significant conclusions can be drawn in terms of PFS and OS compared to the doublet (34), even if to date in clinical practice the bevacizumab-associated triplet is considered a good option for patients with BRAFV600E disease fit for intensive chemotherapy treatment. As far as the association with anti EGFR monoclonal antibodies is concerned, the predictive role of response of patients carrying the BRAF600E mutation is not completely clarified. The data reported in the literature are conflicting, however, to date it is believed that the association of chemotherapy with anti VEGF drugs should be preferred over the combination with anti EGFR (33). The ongoing studies will help to better define the role of target treatments in the first line considering what has been learned from the BEACON study and capitalizing on the main limitations of the target treatment. Once again, the early resistance to treatment due to the complexity of the signal transduction pathways translates into a lower PFS than expected. Further association studies aimed at improving the outcome of this category of patients are underway.

###### 2.1.3.3.1 Future scenario

Starting from the exciting data of BEACON study, as previous mentioned, the combination of the BRAF inhibitor encorafenib plus cetuximab with or without the MEK inhibitor binimetinib is in study in first line. The phase II trial ANCHOR CRC study (NCT03693170) aims to evaluating efficacy, safety, and quality of life of encorafenib + binimetinib + cetuximab in BRAFV600E-mutated mCRC. This study confirmed the expected clinical activity of the combination with a ORR of 47.4%, a mPFS of 5.8 months and a median OS of 18.3 months (35). The BREAKWATER phase 3 trial (NCT04607421) is currently underway to validate the combination plus or minus chemotherapy for the first line treatment (36).

Again, the biological data collected so far from clinical studies will lead to enriching the therapeutic options of patients with the BRAF V600E mutation in the near future. For example, analysis of biopsies from patients enrolled in the BEACON study showed increased T-cell infiltration after initiation of BRAF inhibitor-targeted treatment, suggesting a potential coadjutant between BRAF targeting and immune response, a concept that had already been hypothesized in preclinical studies with the advent of anti BRAF drugs (37). Several phase I/II clinical trials are ongoing to determine the clinical activity of BRAF V600E inhibitor and ICI combinations. Among them, there are several encouraging data as from the phase I study combining dabrafenib-trametinib and spartalizumab which showed a 33% ORR with a 76% of DCR (38), Similar results were published from a SWOG phase I trial demonstrated promising activity of the encorafenib-cetuximab-nivolumab triple combination, with an ORR of 50%, DCR of 95% and a remarkable PFS of 7.4 months (vs 4.2 in the BEACON) and an OS of 15.1 months (vs 9.3 months in the BEACON) (39). It will be interesting to see if the currently ongoing randomized phase II trial (NCT05308446) (40) can confirm these exciting results.

**Figure 2:**
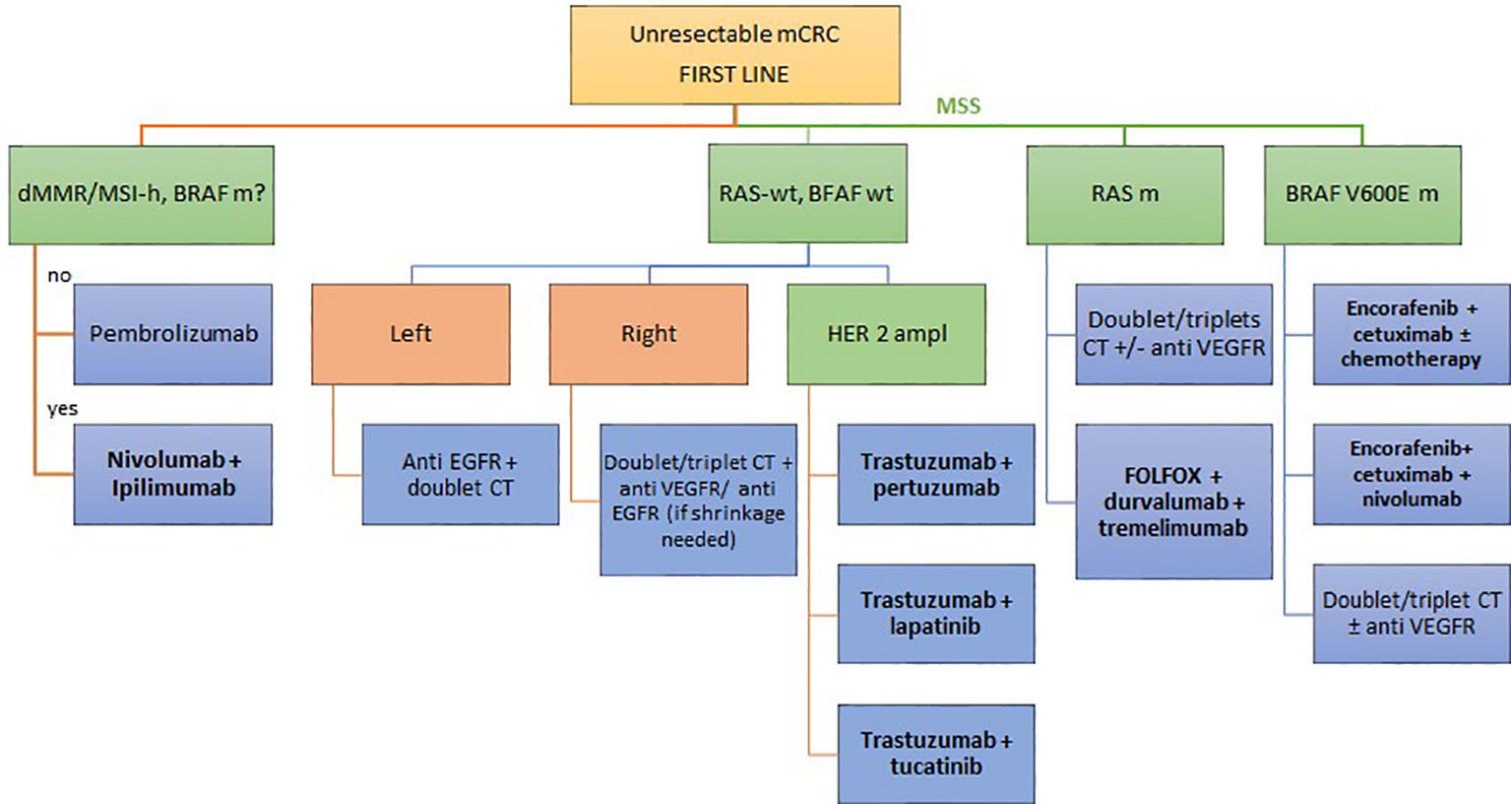
Potential future algorithm for first line mCRC.

The therapeutic scenario can be like this in next future (see **Figure 2**).

## 3 Manteinance therapy

Metastatic patients or patients not eligible for resection in response to first-line therapy continue treatment on maintenance therapy in order to improve treatment tolerance, reducing side effects like peripheral neuropathy, alopecia, gastrointestinal disorders and other side effects without leading to a deterioration in quality of life. However, no clear survival advantage over progression of therapy was demonstrated. In patients with wild type RAS oncogenes, after FOLFOX or FOLFIRI plus cetuximab or panitumumab maintenance strategies with 5-FU + panitumumab resulted to be better than either agent alone (41, 42). However, conflicting data come from the results of the Valentino and Ermes studies. In the first study, maintenance therapy with panitumumab was found to be non-inferior to the continuation of FOLFOX + Panitumumab (43), while in the second trial, PFS was lower in patients treated with cetuximab alone after 8 cycles of FOLFIRI + cetuximab compared to the continuation of the doublet plus anti-EGFR therapy until progression (44). However, in this latter trial, the high dropout rate of patients may have reduced the statistical power of the analysis, but it suggests a PFS and OS benefits associated with cetuximab maintenance therapy. Maintenance strategy with 5FU + anti-EGFR prolonges PFS than observation alone, anti-EGFR monotherapy, and bevacizumab monotherapy (45). This suggest that combination therapy with 5-FU + anti-EGFR agent is effective in this context. Also in RAS,NRAS mutated tumor, anti VEGF monotherapy following induction therapy demonstrated improvement in PFS versus observation (46), however the combination of capecitabine and bevacizumab has a greater impact on PFS than observation (47), bevacizumab monotherapy (48) or stop and go treatment (49). Recent metanalysis confirm that capecitabine with or without bevacizumab is the best option (50). In this review, we only want to mention the possibility of reducing the toxicity of first-line treatment with maintenance therapy that does not harm survival. For further insights, it is recommended to consider dedicated reviews on the topic.

## 4 First-line therapy in patients affected by mCRC MSI-H

Ongoing oncological research and the evolution of cancer molecular biology knowledge have made it clear how the use of biomarkers will have a primary impact in targeted treatments. In this ever-changing scenario there is an important molecular footprint represented by the knowledge of microsatellite instability (MSI), particularly the presence of defects in the mismatch repair system. MSI-H CRC patients account for 10-25% of colon cancer patients, of which 3% are associated with Lynch syndrome, a hereditary form, and the remaining 12% are sporadic caused by hypermethylation somatic and acquired MLH1 gene promoter.

This knowledge changed clinical practice in the adjuvant setting of stage II colon cancer and led to several studies in the neoadjuvant setting with dramatic results from the study conducted by Chalabi and colleagues (51). As regards the first line in the metastatic setting, it has modified clinical practice by introducing the possibility of treatment with immunotherapy in patients affected by MSI-H mCRC, therefore all patients diagnosed with mCRC must be screened for MSI status or by PCR or IHC methods, it is mandatory to identify patients who could benefit from immunotherapy.

The first data on the potential of immunotherapy treatments in mCRC patients with microsatellite instability comes from the phase II clinical conducted by Le and colleagues, who enrolled patients with different cancer histologies, among them 32 patients affected by mCRC of which 11 with MSI-H, showing an objective response to pembrolizumab, a monoclonal anti-PD1 antibody, at 20 weeks of 40% in MSI-H patients (vs. = 5 for MSS patients) and a PFS of 78% in MSI patients (vs. 11% for those with MSS tumors) (52). These results were subsequently confirmed by a phase III study conducted by Andrè and colleagues (KEYNOTE 177) which randomized a total of 307 patients affected by MSI-H mCRC not previously treated with first line in the metastatic setting to receive Pembrolizumab at a dose of 200 mg every 3 weeks or chemotherapy (5-fluorouracil therapy with or without bevacizumab or cetuximab) every 2 weeks. After a median follow-up of 32.4 months, the study concludes with a superior progression-free survival for pembrolizumab compared to chemotherapy (16.5 vs. 8.2 months; 95% confidence interval [CI], P = 0,0002) (53).

These studies were followed by other studies examining the efficacy of anti-PD1 monocolonal antibodies in MSI-H mCRC patients. The study conducted by Overman and colleagues (54) in patients pre-treated with at least 1 line of chemotherapy demonstrated high response rates in patients treated with Nivolumab, anti PD1 monoclonal antibody +/- Ipilimumab (anti CTLA4 monocolonal antibody) suggesting superior efficacy treatment versus PD1 monocolonal antibody alone. These results were then investigated and confirmed in the first line setting with a phase II study conducted by Lenz and colleagues (Checkmate 142) which investigated the activity of Nivolumab in combination with Ipilimumab with very encouraging results. Specifically, of the 45 patients enrolled, 13% had a complete response, 69% (95% CI, 53 to 82) had an objective response to treatment, and there was a disease control rate of 84% (95% CI, 70.5 to 93.5) (55). At present, the Food and Drug Administration (FDA) and the European Medicine Agency (EMA) have approved Pembrolizumab as first-line treatment in patients with MSI mCRC, while the combination Nivolumab and Ipilimumab has been approved by the FDA for subsequent lines of treatment.

Despite the remarkable achievement of introducing immunotherapy as the primary choice for MSI-H mCRC patients, there are two major problems that cancer research has attempted to address and they are mainly based on the following limitations of immunotherapy treatment: firstly the small percentage of patients with mCRC MSI-H (around 5%) which therefore makes a small number of patients eligible for immunotherapy, and secondly the resistance to treatment. The first objective is therefore to increase the proportion of patients susceptible to immunotherapy treatments, through the combination of chemotherapy or biological oncological treatments together with immunotherapy, and the attempt to understand the mechanisms of resistance to treatment with the aim of overcoming them.

### 4.1 Mechanisms of resistance to first line immunotherapy treatment

As overexposed, immunotherapeutic treatments have had a significant impact by modifying a positive clinical history of patients affected by mCRC MSI in terms of overall survival and quickly placing themselves in the indications of the first line of treatment for this category of patients. However, some patients will manifest intrinsic resistance configuring an immunotherapy non-responsive group and others will progress after an initial response thus manifesting acquired resistance to treatment. The percentage of patients resistant to treatment with ICI reported in the literature is around 30%, therefore a significant percentage (56). Clinical studies have focused on combination of treatments with the aim of overcoming these resistances.

The resistance mechanisms best known to date are fundamentally linked to the modification of the tumor microenvironment with reduction of the infiltration of cells of the immune system due to reduced expression of antigens by the disease and the activation of other molecular and oncogenic pathways involved in the growth mechanism such as, for example, angiogenesis (57). Numerous drug combination studies are underway to evade these resistances, and which are therefore associated with anti PDL1 drugs, the most promising include other ICI drugs (such as anti CTLA4) and antiangiogenic drugs. None of these combinations is currently approved for clinical practice in 1st line treatment or after progression to 1st line treatment with ICI.

At present, combinations of chemotherapy are proposed with the possible association of anti-VEGF or EGFR drugs, are proposed for progression to first line with ICI drugs. Data on the clinical response to such chemotherapy regimens after ICI progression are not yet mature and are mostly extracted from retrospective studies (58–60).

## 5 How to overcome ICI resistance

Pembrolizumab and nivolumab + ipilimumab are the regimens currently available for MSI CRC in first line, but unfortunately, almost 30% of MSI CRC resulted primary resistant to ICI. At the basis of these resistances are the development of various mutations, those most studied to date concern inactivating mutations of the kinases of the Janus family (Jaks) such as Jak1 and Jak2, involved in cell growth, survival, and differentiation of various cells especially the cells of the immune system. Jak1 and Jak2 are implicated in both primary and secondary resistance to treatment. Inactivation of other genes involved in MHC class I antigen presentation mechanisms such as the gene coding for microglobulin beta2 protein (B2M), promotes immune escape and consequently a reduction in T cell activation and response to ICI drugs, as well as other genes involved in immune surveillance mechanisms (61).

Several studies have proposed combined treatment regimens to circumvent these resistances or to convert a “cold” tumor into “hot” one. The rationale behind these combinations is represented using drugs which, through tumor cell death, lead to an increase in tumor immunogenicity, alternatively to reverse tumor immunosuppression or to reduce tumor burden by increasing the tumor response.

Many studies are enrolling patients with metastatic colorectal cancer regardless of microsatellite status. Surely these studies will provide an overall view useful for a better understanding of the future therapeutic pathways of patients with mCRC (NCT02375672) (NCT02291289) (NCT03174405). However, it seems that we cannot avoid considering the undeniable differences in terms of treatment outcome of these two pathologies which are in effect two different entities of the same disease.

### 5.1 Combinations of ICI and tyrosine kinase inhibitors

As far as tyrosine kinase inhibitors are concerned, the most studied drug in association with immune checkpoint inhibitor monoclonal antibodies is Regorafenib, a potent oral multikinase inhibitor which has a broad spectrum of activity with inhibition of tyrosine kinases involved in tumor angiogenesis mechanisms (e.g. PDGFR, FGFRs 1–2, VEGFRs 1–3, TIE2), proliferation (e.g. RET, RAF, KIT), tumor microenvironment and metastatic processes (VEGFR2–3, PDGFR). The inhibition of the colony-stimulating factor 1 receptor (CSF-1R) implicated in the regulation of tumor-associated macrophages (TAM) whose role in carcinogenesis, resulting from the inhibition of tumor infiltration by leukocytes, confers to Regorafenib an immuno-modulating role. It is now recognized and documented in various types of tumors. The trials that have evaluated the combination of regorafenib with immune checkpoint inhibitor drugs (ICI) are REGONIVO and REGOMUNE. Encouraging data emerged from the Japanese Phase Ib REGONIVO study investigating the combination of Regorafenib + Nivolumab in patients progressing on two or more lines of treatment showing an ORR of 33% (62) were not confirmed by the Phase II study conducted on an American population (63) which in fact did not meet the primary endpoint. In line with these results also those of the phase II study REGOMUNE which enrolled pretreated MSS mCRC patients to receive regorafenib + Avelumab (64) and of the phase I/II study of combination with Pemrolizumab + Regorafenib (65) which did not register any objective response to treatment. However, considering the REGONIVO phase II study, it is interesting to note that all responder patients had no liver metastases at baseline. If we consider only patients without liver metastases, the ORR rate rises to 22% compared to 7% in the general study population. These fewer encouraging data did not stop research in this sense as the biological rationale, and the data from phase I of the REGONIVO study, had fueled strong hopes. A phase I study is ongoing aiming to enhance this combination with a dual inhibition of the immune checkpoint (NCT04362839), combining regorafenib with ipilimumab and nivolumab in patients with MSS CRC. Preliminary data are encouraging with an ORR of 31%, with a median PFS of 4 months (66). All the studies are collected in **Table 1**.

**Table 1:** Current combination strategies of ICI therapy in CRC.

Strategy	Trial Name/ NCT Number	Phase	Patients (n) Trial Population	Study arms	Results
*Immunotherapy and tyrosine chinase inhibitors*	REGONIVO NCT03406871	Ib	24	Single arm Regorafenib + Nivolumab	ORR: 33% PFS: 7.9 months
	NCT04126733	II	70	Single arm Regorafenib + Nivolumab	ORR: 7% PFS: 1.8 months OS: 12 months
	REGOMUNE NCT03475953	II	48 MSS	Single arm Regorafenib + Avelumab	PFS: 3.6 months OS: 10.8 months
	NCT03657641	I/II	73 (Phase I) 63 (Phase II)	Single arm Regorafenib + Pembrolizumab	mPFS: 2.0 OS 10.9
*Immunotherapy and antiangiogenic drugs*	CheckMate 9 x 8 NCT03414983	II	225	Arm A Nivolumab + SOC Arm B SOC	PFS: 11.9 (A) vs 11.9 (B) months OS: 29.2 (A) vs NR months ORR: 60% (A) vs 46% (B)
	AtezoTRIBE NCT03721653	III	199	Arm A FOLFOXIRI + Beva Arm B FOLFOXIRI + Beva + Atezo	ORR: 59% (A) vs 64% (B) PFS: 12.9 (A) vs 11.4 (B) months
	BACCI NCT02873195	II	82 dMMR	Arm A Capecitabine + Beva + Atezo Arm B Capecitabine + Beva	ORR: 10% (A) vs 5% (B) PFS: 5 (A) vs 3.3 (B) months OS: 10.3 (A) vs 10.2 (B) months
	MODUL NCT02291289	II	445 BRAF wt	Arm A Beva + Fluorouracil + Atezo Arm B Beva + Fluorouracil + Placebo	ORR: 13.8% (A) vs 12.2% (B) mPFS: 7.2 (A) vs 7.4 (B) months OS: 22 (A) vs 22 (B)
	POCHI NCT04262687	II	55 High immune infiltrate MSS	XELOX + Beva + Pembrolizumab	Recruiting Estimanted Study Completion date: 30 Sep 2024
	COMMIT NCT02997228	III	120 dMMR/MSI-H	Arm A Atezolizumab Arm B FOLFOX + Bevacizumab + Atezolizumab	Recruiting Estimanted Study Completion date: 30 Nov 2024
*Immunotheraphy and EGFR inhibitors*	CAVE NCT04561336	II	71 RAS wt	Single arm Avelumab + Cetuximab	ORR: 8.5% PFS: 3.6 months OS: 11.6 months
	AVETUX-CRC NCT03174405	II	43 RAS/BRAF wt	Single arm Avelumab + Cetuximab + mFOLFOX6	ORR: 79.5% PFS: 11.5 months
*Immunotherapy and MEK inhibitors*	NCT03428126	II	29 MSS	Single arm Durvalumab + Trametinib	ORR: 3.4% mPFS: 3.2 months
	NCT01988896	Ib	59	Single arm Atezolizumab + Cobimetinib	ORR: 8% mPFS: 1.9 months OS: 9.8 months
	IMblaze 370 NCT02788279	III	183	Arm A Atezolizumab + Cobimentinib Arm B Atezolizumab Arm C Regorafenib	OS: 8.87 (A) vs 7.10 (B) vs 8.51 (C)
	NCT03271047 (C4211004)	Ib/II	42/48 MSS, RAS mut	Arm A Binimetinib + Nivolumab Arm B Binimetinib + Nivolumab + Ipilimumab	ORR: 0% (A) vs 7.4 (B)
	NCT03475004	II	53 MSS	Single arm Pembrolizumab + Bevacizumab + Binimetinib	ORR: 13% mPFS: 5.8
*Double immunotherapy or immunotherapy and chemotherapy*	CheckMate-142 NCT02060188	II	23 MSI-H/dMMR	Cohort 3: Nivolumab + Ipilimumab	PFS: 1.4 months
	MEDITREME NCT03202758	I/II	57 RAS mut	Single arm Durvalumab + Tremelimumab + FOLFOX	PFS: 8.4 months ORR: 61%
	C-800 NCT03860272	I	41 MSS	Single arm Botensilimab + Balstilimab	ORR: 24%
	NCT0560844	II	230	Cohorts 1-2 Botensilimab + Balstilimab Cohorts 3-4 Botensilimab at different doses Cohort 5 SOC	Recruiting
	NCT02860546	II	18 MSS	Single arm Trifluridine/tipiracil + Nivolumab	ORR: 0% PFS: 2.8 months
	NCT03832621	II	33 MSS, MGMT-silenced	Single arm Temozolamide + Nivolumab + Ipilimumab	ORR: 45% PFS: 7 months OS: 18.4 months
	ARETHUSA NCT03519412	II	102 MSS, MGMT IHC-negative	Arm A Temozolamide + Pembrolizumab Arm B Pembrolizumab	Recruiting

### 5.2 Combinations of ICI with antiangiogenic drugs

The combination of cytotoxic agents and bevacizumab in colorectal cancer could enhance the efficacy of immune drugs by increasing neoantigen exposure, especially with highly active chemotherapy regimens, inducing immune-mediated cell death, increasing tumor lymphocyte infiltration CD8+ T cells and reducing tumor-associated myeloid-derived suppressor cells. VEGF blockade also plays an immunomodulatory role by blocking the expansion of regulatory T lymphocytes. Based on this rationale, combination studies with bevacizumab chemotherapy and ICI were performed. The Phase II CheckMate 9 × 8 study (NCT03414983) compared the addition of nivolumab to FOLFOX and bevacizumab. The ORR was 60% in the experimental group and 46% in the standard of care control, longer responses, and acceptable safety, however the study did not meet the primary endpoint of PFS (67). This data was confirmed also from AtezoTRIBE study which randomized mCRC patients to receive standard (FOLFOXIRI and bevacizumab) or investigational (FOLFOXIRI, bevacizumab and atezolizumab). The study failed to demonstrate significant improvement in mPFS (12.9 m vs. 11.4 m, p = 0.072) (68). The BRACCI study evaluating the combination of capecitabine+bevacizumab with or without atezolizumab in chemo-refractory setting regardless of MSS status met its primary endpoint of median PFS (4.4 vs 3.6 months) in the overall population with nonsignificant improvement in PFS in the MSS subgroup (5.3 vs 3.3 months) (69). The MODUL study evaluates the combination of bevacizumab + fluorouracil with or without the addition of atezolizumab in selected BRAF wild type patients. Four hundred and forty-five patients were randomized in this study, however at a median follow-up of 20 months, the PFS benefit was not achieved (HR 0.95; 95% CI 0.77-1.18; P = 0.666) (70).

A study is underway in the first-line setting (POCHI TRIAL) which is evaluating the association of pembrolizumab in combination with Xelox and bevacizumab in patients with MSS mCRC with a high immune infiltrate. The presence of a high lymphocytic infiltrate is evaluated on blocks of resected primary tumor and analyzed prospectively. For each patient, slides containing tumor tissue and adjacent non-tumor tissue will be analyzed using two techniques: immunoscore^®^ and TuLIS score, consisting in immunohistochemistry with CD3 and CD8 staining. Tumors will then be classified as having a “high” or “low” immune response according to type of lymphocyte infiltrate, which is independent of pre-analytic conditions. Only patients with a high immune response will be eligible for the trial (NCT04262687) (71).

In order to overcome primary immunotherapy resistance in MSI patients the phase III COMMIT study randomized previously untreated MSI-H/MMRD mCRC patients into 2 arms: atezolizumab + FOLFOX/Bevacizumab or a monotherapy arm with atezolizumab (72). The primary endpoint is PFS, and the results are hugely awaited. All the studies are collected in **Table 1**.

### 5.3 Combinations of ICI with EGFR inhibitors

Combinations of ICI with chemotherapy and anti-EGFR drugs have been investigated in several trials in patients with MSS mCRC and wild type RAS status. Already from the first studies that investigated the activity of anti EGFR drugs, an immunomodulatory role of these molecules had been hypothesized through additional antitumor activity including FcγRs-mediated antibody-dependent cellular cytotoxicity, phagocytosis, and T cell-mediated immune response (73). At the basis of this rationale, the combinations of ICI drugs with schemes based on chemotherapy and anti EGFR monocolonal antibodies were therefore studied. Among these are numerous studies in treatment-experienced patients, such as the single-arm phase II CAVE trial combining avelumab + cetuximab in a pretreated and chemorefractory population (74). Focusing on first line trials, the phase II Avetux study (NCT03174405) investigated the activity of Avelumab in combination with FOLFOX+ Cetuximab in patients with previously untreated RAS/BRAF wildtype mCRC independent of MSI status. This study concluded with very promising data recording an ORR of 79.5%, including 6 complete responses (CR) and 25 partial responses (PR). PFS of 11.1 and OS of 32.9 months. The early tumor shrinkage (ETS) rate (≥20% after 8 weeks) was 79.5% (75). Once again, the translational data are of fundamental importance to better understand the data relating to the clinical response of the treatments. In particular, the best radiological response was evaluated in the light of potential response biomarkers of immune checkpoint blockade and showed that clonality and diversity, but not frequency of tumor infiltrating lymphocytes (TiLs) and peripheral blood mononuclear cells (PBMCs), were strongly correlated with response. Thus, proposing T-cell clonality and diversity as a potential marker to predict response to chemo-immunotherapy combinations in mCRC with MSS. All the studies are collected in **Table 1**.

### 5.4 Combinations of ICI with MEK inhibitors

Many studies have investigated the clinical activity of the combination of immune checkpoint inhibitor drugs and MEK inhibitors (MAP kinase inhibitors) since the MAP (Mitogen-activated protein) kinase pathway is one of the signal transduction pathways most involved in the colorectal cancer carcinogenesis process (76). This pathway is also involved in the mechanisms of immunosuppression and reduced tumor infiltration of T cells, accordingly the presence of the MAP1K2 mutation is reported in the literature as a predictor of response to ICI drugs in patients with melanoma (77). The strong rationale had raised hopes that such a combination was the key to evading the low immunogenicity of MSS mCRC diseases (78). However, the combinations studied to date in clinical trials have not given the desired results in terms of response to treatment in a setting of previously treated patients (79–81). What emerges from these studies is that probably in colorectal tumors, the single block of MEK fails to maintain the inhibition of the MAP kinase pathway, probably due to the activation of adaptive feedback mechanisms, such as the EGFR receptor. Phase II second-line trials combining MEK inhibitors with dual immune blockade (NCT03271047) (82) and anti-PD1+ antiangiogenic drugs (NCT03475004) (83) are currently underway. All the studies are collected in **Table 1**.

### 5.5 Combinations of double ICI or ICI plus chemotherapy

Data known until recently about the use of double inhibition of the immune checkpoint in patients with MSS disease was not encouraging. Among these, the phase II CheckMate-142 study, which led to the approval of nivolumab+ipilimumab in patients with MSI-H disease, also enrolled 23 patients with stable disease to receive Nivolumab (anti-PD1) + ipilimumab (anti-CTLA4) which ended with a median PFS of 1.4 months in this category of patients suggestimg a lack of clinical activity of combined blockade of PD-1 and CTLA4 in mCRC MSS (55). However, these disappointing results have not stopped clinical research in which other combinations of immunotherapy have been attempted, with or without chemotherapy regimens.

As for the former, in the phase II MEDETREME trial, patients with MSS-mutated KRAS mCRC were enrolled to receive mFOLFOX6 (6 cycles) in combination with durvalumab (150 mg/q2W) and tremelimumab (75 mg/q4W). After 6 cycles of chemotherapy, the cancer treatment continued with durvalumab until progression. The primary endpoint was the 6-month progression-free survival rate. The median PFS was 8.4 months. Translational data from the study aimed to identify which category of patients could benefit from such a therapeutic combination and showed that high baseline MDSC Th2 and PDL1+ levels were associated with poor PFS (84). As regards instead the double pure immune blockade, without the addition of chemotherapy, the recent data on the activity of the combination of new ICI drugs is very interesting. In particular, the C800 study presented at Esmo GI 2022, combined Botensilimab, a novel innate/adaptive immune activator (Fc-enhanced CTLA4 inhibitor) with Balstilimab (an anti-PD1). In this study, 41 heavily pretreated MSS CRC patients were enrolled to receive dual immune blockade, recording an impressive ORR of 24% and a disease control rate (DCR) of 73%, with a good safety profile. Once again, the presence of liver metastases seems to affect the clinical response to treatment. Indeed, exploratory analysis showed higher responses in the proportion of patients without active liver metastases (ORR 42%, DCR 96%) again suggesting that liver metastases may somehow preclude resistance to ICI treatment in MSS disease (85). Given these significant data, FDA has granted fast track designation for the combination of botensilimab with balstilimab in patients with MSS mCRC. This combination will be evaluated in a phase III study (NCT05608044) planned to launch in 2023.

Combinations with chemotherapy drugs were evaluated in both polychemotherapy and monochemotherapy schemes. As regards monochemotherapy combinations, these are mostly studies in settings of heavily pretreated patients, such as for example the study which evaluated the addition of Nivolumab to oral treatment with TAS102. This study did not demonstrate any clinical benefit of this combination (86). As far as first line studies are concerned, several studies are evaluating the combination of ICI drugs with Temozolamide, a chemotherapy used in glioblastoma patients, which is thought to have an immunomodulatory role. In particular, the phase II Maya study evaluated the combination of temozolomide priming followed by a combination of low-dose ipilimumab and nivolumab in patients with MSS and O6-methylguanine-DNA methyltransferase (MGMT)-silenced mCRC. At a median follow-up of 23.1 months the Maya study concludes with an 8-month PFS rate was 36% and the overall response rate was 45%, providing evidence that a priming sequence with Temozolomide followed by a combination of low-dose ipilimumab and nivolumab may induce sustained clinical benefit in MSS- and MGMT-silenced mCRC (87). Similar Phase 2 Study is also evaluating for First-Line Treatment in MSS Patients with MGMT IHC-negative mCRC Pembrolizumab in combination with Temozolamide (88). All the studies are collected in **Table 1**.

## 6 mCRC with Her2 and other mutations

With the advent of next generation sequencing (NGS), a greater understanding of the mutational status of colorectal tumors is emerging with a focus on some mutations which, albeit with low incidence, could be potential therapeutic targets. The most promising trials are illustrated below.

### 6.1 Over expression of Her2 oncogene

The results obtained in targeting her2 in other tumors such as breast, skin, gastric cancers, together with the knowledge of a certain incidence of her2 overexpression in mCRC tumors (about 2%), has led clinical research to study the efficacy data of the anti-her2 target treatment in this category of patients. At the present time, data are immature and will have to be investigated on a larger patient population, a very difficult undertaking given the small percentage of patients who carry this mutation. The HERACLES-A evaluated the combinations of trastuzumab in combination with lapatinib, a dual HER1/HER26 tyrosine kinase inhibitor in HER2+ pretreated patients in the metastatic setting concluding with an ORR of 30%, and a surprising median survival (10 months) considering the heavily pretreated population (89, 90). These results led to the inclusion of trastuzumab and lapatinib regimen in the 2019 NCCN Guidelines for mCRC. Other clinical trials followed, such as for example the HERACLES-B study enrolled 31 heavily pretreated patients in the metastatic setting to receive the combination of Pertuzumab and trastuzumab emtamsine (TDM1). Most patients had received more than 4 prior lines of treatment, and this may have impacted expected ORR which failed to meet the primary endpoint of the study. In particular, the ORR was 9.7% (95% CI: 0 to 28) and stable disease (SD) was 67.7% (95% CI: 50 to 85). It is interesting to underline that OR/SD ≥4 months was associated with higher HER2 immunohistochemistry score (3+ vs 2+) (p = 0.03) and this data could help in the better selection of candidates for such combinations in subsequent clinical trials (90). Consistent data has been published from the MyPathway study with the trastuzumab-pertuzumab combination (91). However, the setting in which these associations have been investigated is always a setting of heavily pretreated patients with at least 2 lines of treatment (92–96). At present, therefore, we do not have first-line data, therefore a target anti-her2 treatment is feasible in this setting only for patients not eligible for standard first-line treatments. Since there are no ongoing trials in the first line setting, it is difficult to conceive a target anti-her2 treatment in this setting in the short term.

### 6.2 NTRK gene fusions, RET, ROS1

There are other targetable mutations identifiable in a small fraction of mCRC patients, including NTRK, ROS1, ALK, RET fusions. The NCCN guidelines contemplate the use of specific target treatments in patients harboring these mutations in a non-first-line metastatic setting. Strategies targeting gene fusions is an attractive field of research, however, in most countries patients do not have access to comprehensive molecular profiling through NGS. Considering for example the fusion of NTRK, present in less than 1% of patients with mCRC, at present it is not sought in all patients but in selected cases and after discussion of the case within the molecular tumor board, where present. Currently two drugs have received FDA and EMA approval for the treatment of patients with mCRC and NTRK fusion for non-first-line treatment, Larotrectinib and Entrectinib, both based on small phase I and II studies designed as tumour-agnostic basket trials (LOXO-TRK -14001, SCOUT and NAVIGATE, ALKA-372-001, STARTRK1, STARTRK-2 and STRATRK-NG) whose combined analysis demonstrated a rapid and durable response rate with a good toxicity profile. One of the most important challenges for future medical oncology will be to guarantee access to targeted treatments from the point of view of precision medicine, which will become an unavoidable urgency to ensure good quality care. At the same time, understanding the mechanisms of treatment resistance will be of considerable importance to ensure maintenance of treatment response. Clinical trials of new NTRK fusion inhibitor drugs are underway that should circumvent treatment resistances with Larotrectinib and Entrectinib that are mostly due to the development of mutations that decrease binding affinity of NTRK inhibitors to the kinase domain and the downstream activation of MAPK signaling (97). The second-generation NTRK inhibitors selitrectinib (LOXO-195) and repotrectinib (TPX-0005) are currently in Phase I/II trials (98, 99).

## 7 Discussion

Over the years, the complexity of colorectal cancer has become increasingly apparent as our understanding of the disease’s biology has grown. Initially, we classified colorectal cancer based on its site of origin, distinguishing between right and left colon, and considered prognostic and treatment response factors. Subsequently, we started examining the presence of mutations, which in turn influenced prognosis and treatment responses. The discovery of distinct responses to fluoropyrimidine-based treatments in patients with microsatellite instability led to the emergence of immunotherapy. Consequently, a more intricate landscape has emerged, necessitating the codification of various therapeutic options. Therefore, opting for a therapeutic strategy targeting a single aspect no longer appears suitable for treating colorectal cancer.

Starting with tumors originating in the left colon with wild-type characteristics, they benefit from anti-EGFR treatments. However, recent evidence has revealed that a deeper understanding of different KRAS mutations has unveiled new therapeutic targets. Currently, combinations of KRAS G12C inhibitors with anti-EGFR antibodies show the most promise (24, 25), generating considerable anticipation for the results of the randomized phase III trial (NCT04793958). Research into the best combinations of KRAS inhibitors with other drugs is thriving, including studies targeting more common KRAS mutations in colorectal cancers, such as KRAS G12D. The primary challenge lies in collecting data on the toxicity profile of these combinations and identifying the patients who stand to benefit from them.

Considering the significant benefits of immunotherapy for patients with microsatellite instability, scientific efforts are focused on expanding the pool of eligible patients for these treatments. However, numerous studies conducted in this context emphasize the crucial importance of selecting patients correctly for immunotherapy. Whether it involves assessing a high immunoscore, as indicated in the Atezotribe study (68), or evaluating the clonality and diversity of tumor-infiltrating lymphocytes, as suggested by the AVETUX study (75), specific selection criteria tied to the disease’s immunogenicity level are required to identify patients who may benefit from immune checkpoint inhibitor combinations, even if they have stable microsatellite status.

Another factor to consider when selecting patients for combination therapies with immune checkpoint inhibitors could be the distribution of the disease. Some studies, including REGONIVO, demonstrate how the presence of liver metastases may contribute to therapy resistance, possibly due to alterations in the microenvironment of this metastatic site.

It is crucial to leverage insights gained from second-line studies to design new first-line treatment options for metastatic patients, offering quicker access to effective treatments in a more favorable disease context. This is especially important given that the percentage of patients responding to oncological treatments significantly decreases beyond the first-line setting. Once again, the insights from translational biology must inform the design of future studies. Data collected from biopsies of patients participating in the BEACON study suggest increased T-cell infiltration after initiating Dabrafenib, hinting at a potential synergy between BRAF targeting and the immune response. This concept underlies promising new clinical trials combining immune checkpoint inhibitors and BRAF inhibitors in the first-line setting (39, 100).

While there is limited and preliminary clinical trial data on other potential mutations in metastatic colorectal cancer, each of these studies can provide valuable insights into the biology of colorectal cancers. Understanding these mutations is urgently needed to better select patients and improve outcomes in this disease. This has a profound impact on individual health and public health. Unlike some other cancers, such as lung cancer, where targeted therapies have quickly transitioned to first-line treatments due to their demonstrated benefits over standard chemotherapy, the major obstacle to introducing first-line targeted therapies in metastatic colorectal cancer is the feasibility of conducting randomized studies comparing these therapeutic options to the current standard of first-line treatment, primarily due to the small number of patients with these mutations. Consequently, it is challenging to foresee the rapid adoption of first-line target treatments for NTRK fusion, ROS1, RET, or HER2 mutations. The scientific community is increasingly focused on finding the most effective first-line treatment to reduce disease burden, potentially enabling the removal of residual disease, improving prognosis, and enhancing patients’ quality of life.

## 8 Conclusion

Interesting novelties and therapeutic opportunities are emerging in mCRC tumors, a pathology in which it has always been difficult to significantly affect patient outcomes and where target treatments had given disappointing results so far. Greater understanding of the biology of this disease, together with enthusiastic clinical research aimed at increasing the proportion of patients eligible for effective and personalized target treatments, will lead to impactful changes in the first-line treatment in the near future.

## Author contributions

Study concept and design: AZ, SC, EO. Drafting of the manuscript: EO, SC. Drafting of the manuscript: SC. Critical revision of the manuscript for important intellectual content: EO, AZ. Study supervision: AZ. Figures and tables: EO, LZ. All authors contributed to the article and approved the submitted version.
